# Alyssum (*Lobularia maritima*) selectively attracts and enhances the performance of *Cotesia vestalis*, a parasitoid of *Plutella xylostella*

**DOI:** 10.1038/s41598-020-62021-y

**Published:** 2020-04-15

**Authors:** Yanting Chen, Jun Mao, Olivia L. Reynolds, Wenbin Chen, Weiyi He, Minsheng You, Geoff M. Gurr

**Affiliations:** 1 State Key Laboratory of Ecological Pest Control for Fujian and Taiwan Crops, Institute of Applied Ecology, Fujian Agriculture and Forestry University, Fuzhou, 350002 China; 2 Joint International Research Laboratory of Ecological Pest Control, Ministry of Education, Fuzhou, 350002 China; 3 Key Laboratory of Green Pest Control (Fujian Agriculture and Forestry University), Fujian Province University, Fuzhou, 350002 China; 4 Fujian-Taiwan Joint Centre for Ecological Control of Crop Pests, Fujian Agriculture and Forestry University, Fuzhou, 350002 China; 5 Graham Centre for Agricultural Innovation, Charles Sturt University, Orange, New South Wales 2800 Australia; 6 cesar, 293 Royal parade, Parkville, Victoria, 3052 Australia

**Keywords:** Agroecology, Behavioural ecology, Animal behaviour

## Abstract

The use of nectar-providing plants to nourish natural enemies of pest species has become a widely-used approach in conservation biological control to reduce pest damage without the indiscriminate use of insecticides. Choice of plant species is crucial to maximize benefits, but suitable species are yet to be identified for many important crop-pest systems. Here we explored the suitability of three candidate nectar plants for use in brassica vegetables to suppress the globally significant pest, *Plutella xylostella* L. (Lepidoptera: Plutellidae), using the widely-distributed parasitoid, *Cotesia vestalis* (Haliday) (Hymenoptera: Braconidae). Volatiles of alyssum (*Lobularia maritima* (L.) Desv) (Brassicaceae) were attractive to the parasitoid and access to flowering shoots increased adult longevity and realized fecundity of *C. vestalis*. Moreover, adult diamondback moth derived no benefit from this flower. In contrast, buckwheat (*Fagopyrum esculentum* Moench) (Polygonaceae), a species widely used in conservation biological control in other systems, increased the longevity and fecundity of both pest and parasitoid, rendering it less suitable. A third plant, heronsbill (*Portulaca grandiflora* Hook.) (Portulacaceae) denied benefit to the pest and promoted longevity of the parasitoid under no-choice conditions but did not improve fecundity and was repellent to female parasitoids under choice conditions. The contrasting effects of this set of plants illustrate the need to test multiple response variables and effects on both pest and natural enemy when seeking optimal nectar plants for use in a novel conservation biological control system.

## Introduction

Intensive agricultural monocultures and simple agricultural landscapes do not provide natural enemies with favorable resources and conditions^1–3^. Natural enemy populations are negatively affected by frequent and intense human disturbance from harvest, tillage and pesticide use that characterize intensive agriculture^2,4^. Remnant non-crop habitats such as ground cover or wild plants can provide shelter, being a source habitat from which natural enemies can colonize crops^1,5^. The reintroduction of appropriate flowering plants into monocultures can promote longevity, fecundity, sex ratio and searching capacity of natural enemies such as parasitoids^6–9^. The addition of flowering plants within or around crop fields can enhance the impact of natural enemies by providing nectar, pollen^10–12^, alternative hosts or prey^13^, favorable microclimatic conditions^14^ and shelter^1,15,16^. Nectar from flowering plants is an important supplementary food which can increase parasitoid longevity and favor biological control^17,18^.

Providing natural enemies with flower-produced resources such as nectar poses the risk of also provisioning pests with such resources, which potentially can aggravate the pest problem^9,19,20^. Most adult Lepidoptera feed on nectar^19^ resulting in an increase of longevity and fecundity of pests. Examples include the Lepidopteran pests *Phthorimaea operculella* (Zeller) (Gelechiidae)^20^, *Pieris rapae* (L.) (Pieridae)^21,22^ and also *Plutella xylostella* (L.) (Plutellidae)^21^. Therefore, it is vital to identify suitable plant species that selectively benefit the third trophic level including parasitoids, without improving the fitness of pests.

The success of utilizing nectar sources of parasitoids depends on several factors including parasitoid mouthpart morphology, floral morphology, flower color, which, in turn, can affect flower attractiveness^23–27^. Flowering plant odor is known to play a crucial role in the food-foraging behavior of parasitoids^28^. Kugimiya *et al*.^29^ reported that female *Cotesia vestalis* (=*plutellae*) (Haliday) (Hymenoptera: Braconidae) can orientate to nearby flowers of *Brassica rapa* L. (Brassicaceae) using olfactory cues. Nectar contains a broad spectrum of secondary metabolites, including volatiles^30^. Flower volatiles can enhance visitor attraction^31,32^, but they may also repel parasitoids^33^. Laboratory screening of certain flowers that enhance the performance of parasitoids are typically performed in cages^34–36^. The primary criterion by which flower species are selected is the relatively easily measured parameter of parasitoid adult longevity^7^ though some studies extend to assess realized fecundity, sex ratio or searching behaviour^6,8,9^ and some have investigated potential unwanted benefits to pests^9^. Although the enhanced performance indicated that parasitoids can acquire the nectar from flowers, it can not guarantee that parasitoids will visit the flowers in the field^25^. Therefore, the attractiveness of floral resources is an important parameter that needs to be considered.

The diamondback moth (DBM), *P. xylostella*, is one of the most important pests in cruciferous plants worldwide, causing US$4-5 billion in crop loss and control costs each year^37–39^. Insecticide application is the main control method used to suppress *P. xylostella* populations, which has led to the development of resistance of *P. xylostella* to most insecticides^40,41^. Insecticides also have negative effects on non-target natural enemies including the parasitoid *C. vestalis*^42^. *C. vestalis*, a specialist larval endoparasitoid of *P. xylostella*, is reported to be a powerful biological control agent against *P. xylostella*^37,43–45^. This parasitoid is widely distributed in China, Europe, South Africa, Pakistan, India and Indonesia and introduced to many regions for biological control in Australia, Commonwealth of Dominica, Fiji, Thailand, the United States and St. Helena (reviewed by Sarfraz *et al*.^46^). It attacks the second and third instar larvae of *P. xylostella*, with host location aided by volatile organic compounds emitted by plants infested with larvae of *P. xylostella*^47^. *C. vestalis* is a synovigenic parasitoid, i.e. the female emerges with few mature eggs^48^, so they need to search for supplemental foods – principally nectar – to obtain energy and nutrients for egg maturation and energy for foraging^49,50^. Without sugar, honey or nectar as supplemental foods, *C. vestalis* females survived only a few days^29,51,52^.

Lack of supplemental food for *C. vestalis* in the field and intensive farming practices make it valuable to develop conservation biological control strategies to increase its efficiency against *P. xylostella*. Therefore, the aim of this study was to identify suitable flowering plants that can selectively attract and enhance the performance of *C. vestalis* without benefiting *P. xylostella*. In our study, we tested three flowering plants under laboratory conditions, including a long-inflorescence, heronsbill (*Portulaca grandiflora* Hook.) (Portulacaceae), a perennial that can grow under adverse conditions^53^, alyssum (*Lobularia maritima* (L.) Desv) (Brassicaceae) and buckwheat (*Fagopyrum esculentum* Moench) (Polygonaceae). Buckwheat is a commercial crop and commonly used in other conservation biological control systems. Alyssum also is widely used in conservation biological control efforts and offers the advantage over buckwheat of a longer flowering period. Heronsbill is a hardy species that could readily be established in field margins and, like alyssum, flowers for an extended period. The hypotheses of the present study were that (1) flowering plants differ in the extent of benefit afforded to *P. xylostella* and *C. vestalis*; (2) the attractiveness of flowering plants does not necessarily correlate with the performance of *C. vestalis* on these flowering plants.

## Results

### Effect of flowering plants on *C. vestalis* adult longevity and fecundity

**Figure 1:**
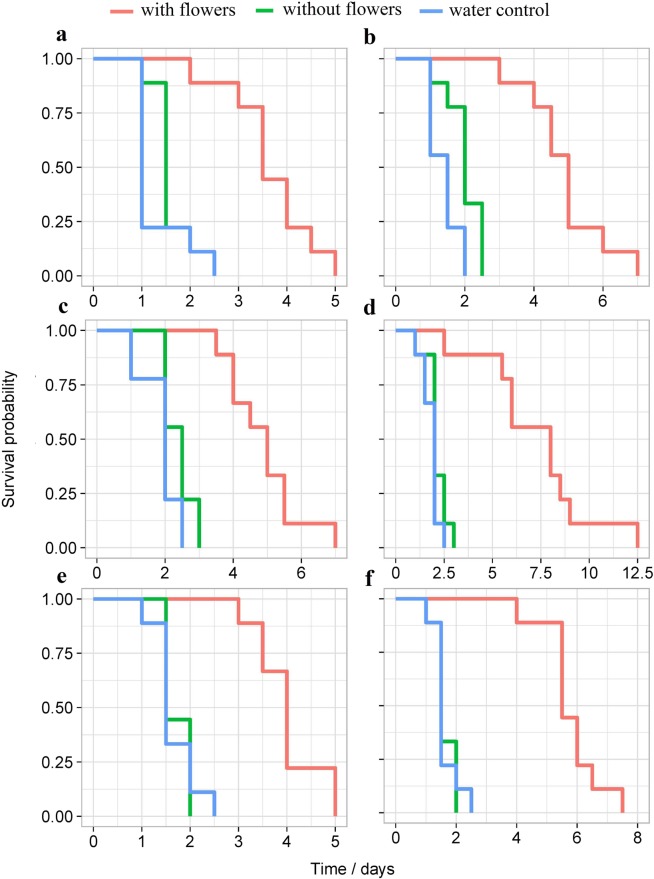
Kaplan-Meier estimates of survival functions for male (**a,c,e**) and female (**b,d,f**) *C. vestalis* with different flowering plants. (**a,b**) *P. grandiflora* (heronsbill); (**c,d**) *F. esculentum* (buckwheat); (**e,f**) *L. maritima* (alyssum).

All plants with flowers tested in this experiment significantly increased the adult longevity of both male and female *C. vestalis* compared with plants without flowers and the water control. Female *C. vestalis* survived for up to 12.5 d with buckwheat flowers, and this longevity was about seven fold superior to water alone (Fig. 1). When provided access to alyssum flowers, the mean female and male adult longevity of *C. vestalis* (5.8 d and 4.0 d respectively), were significantly greater compared to water only and plants without flowers (Kruskal-Wallis test: female: χ^2^ = 18.765, df = 2, *P* < 0.001; male: χ^2^ = 18.652, df = 2, *P* < 0.001). Females presented heronsbill flowers lived significantly longer than males (one way ANOVA: F_2, 26_ = 6.563, *P* = 0.021). Similarly, females lived longer than males on buckwheat (one-way ANOVA: F_2, 26_ = 6.069, *P* = 0.025) and alyssum flowers (one-way ANOVA: F _2, 26_ = 21.558, *P* < 0.001).

**Figure 2:**
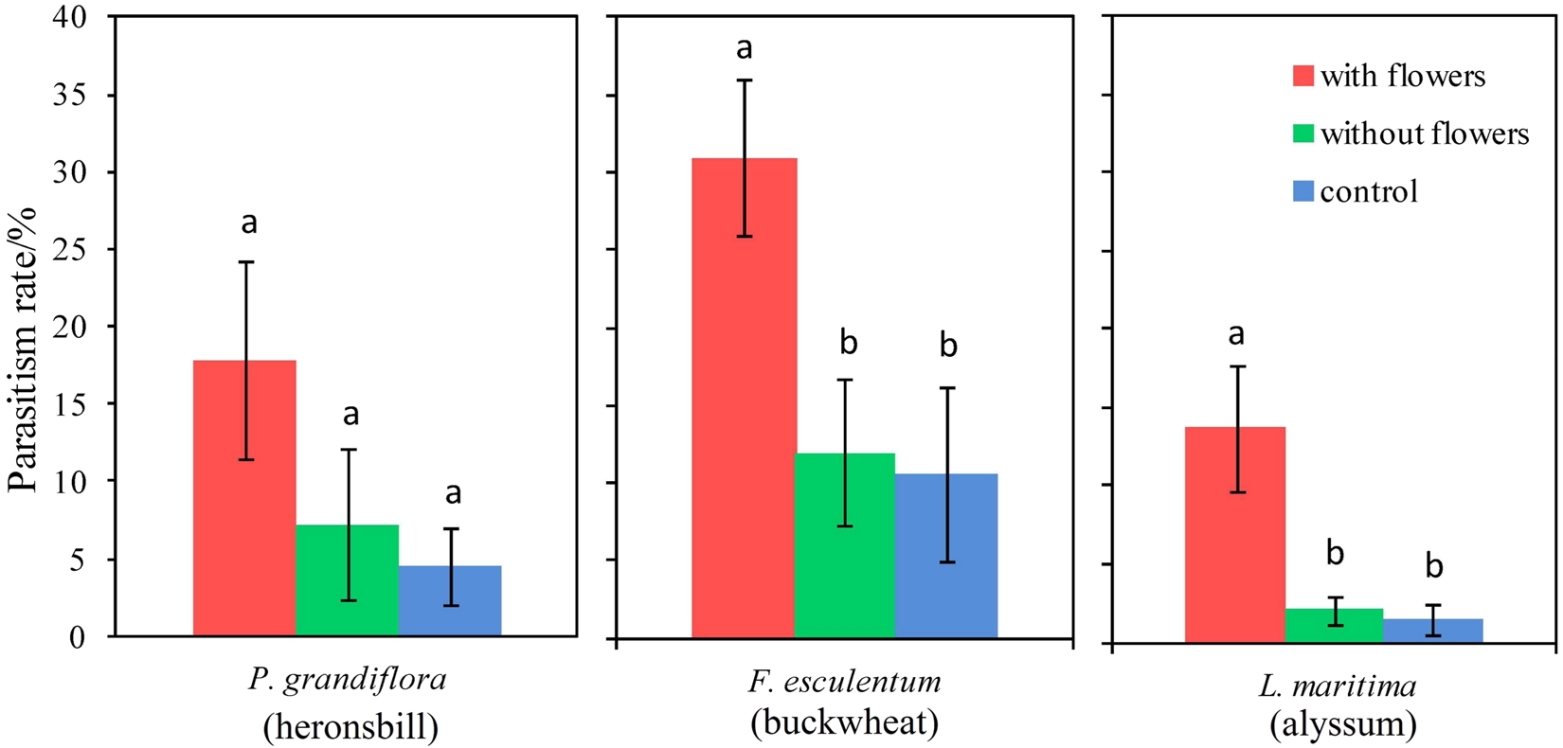
Percentage parasitism by *C. vestalis* with plants with flowers, plants without flowers and water only. Treatments within flowering plant species labeled with the same letter are not significantly different (Kruskal-Wallis test, α < 0.05), error bars = ±1 SE (one pair of *C. vestalis* adults in each treatment, 9 replications).

Buckwheat with flowers significantly increased parasitism of *P. xylostella* by *C. vestalis* to 30.97 ± 5.09%, compared to plants without flowers (11.94 ± 4.72%) and water only (10.60 ± 5.59%) (Kruskal-Wallis test: χ^2^ = 6.314, df = 2, *P* = 0.043). Similarly, realized parasitism (13.68 ± 4.03%) for *C. vestalis* was enhanced by alyssum flowers (Kruskal-Wallis test: χ^2^ = 9.254, df = 2, *P* = 0.01; Fig. 2). In contrast, realized parasitism of *C. vestalis* was not markedly different between heronsbill with flowers, heronsbill without flowers and the water control (Kruskal-Wallis test: χ^2^ = 4.884, df = 2, *P* = 0.087).

### Effect of flowering plants on *P. xylostella* adult longevity and fecundity

Both male and female *P. xylostella* attempted to feed on all three flowering plant species. Mean female and male longevity of *P. xylostella* were 7.44 ± 0.82 d and 8.11 ± 1.06 d respectively in the presence of buckwheat flowers, significantly longer than without flowers treatment (3.50 ± 0.19 d and 4.44 ± 0.60 d) and the water control (3.78 ± 0.29 d and 4.22 ± 0.43 d) (Welch ANOVA: female: F = 10.449, *P* = 0.002; one-way ANOVA: male: F_2, 26_ = 8.573, *P* = 0.002). The availability of heronsbill flowers did not impact the longevity of *P. xylostella* compared to other treatments for either females (Kruskal-Wallis test: χ^2^ = 3.866, df = 2, *P* = 0.145) or males (Kruskal-Wallis test: χ^2^ = 4.786, df = 2, *P* = 0.091). Similarly, the presence of alyssum flowers did not significantly increase the longevity of female and male adult *P. xylostella *(female: Kruskal-Wallis test: χ^2^ = 3.591, df = 2, *P* = 0.166; male: Welch ANOVA: F = 2.373, *P* = 0.127).

**Figure 3:**
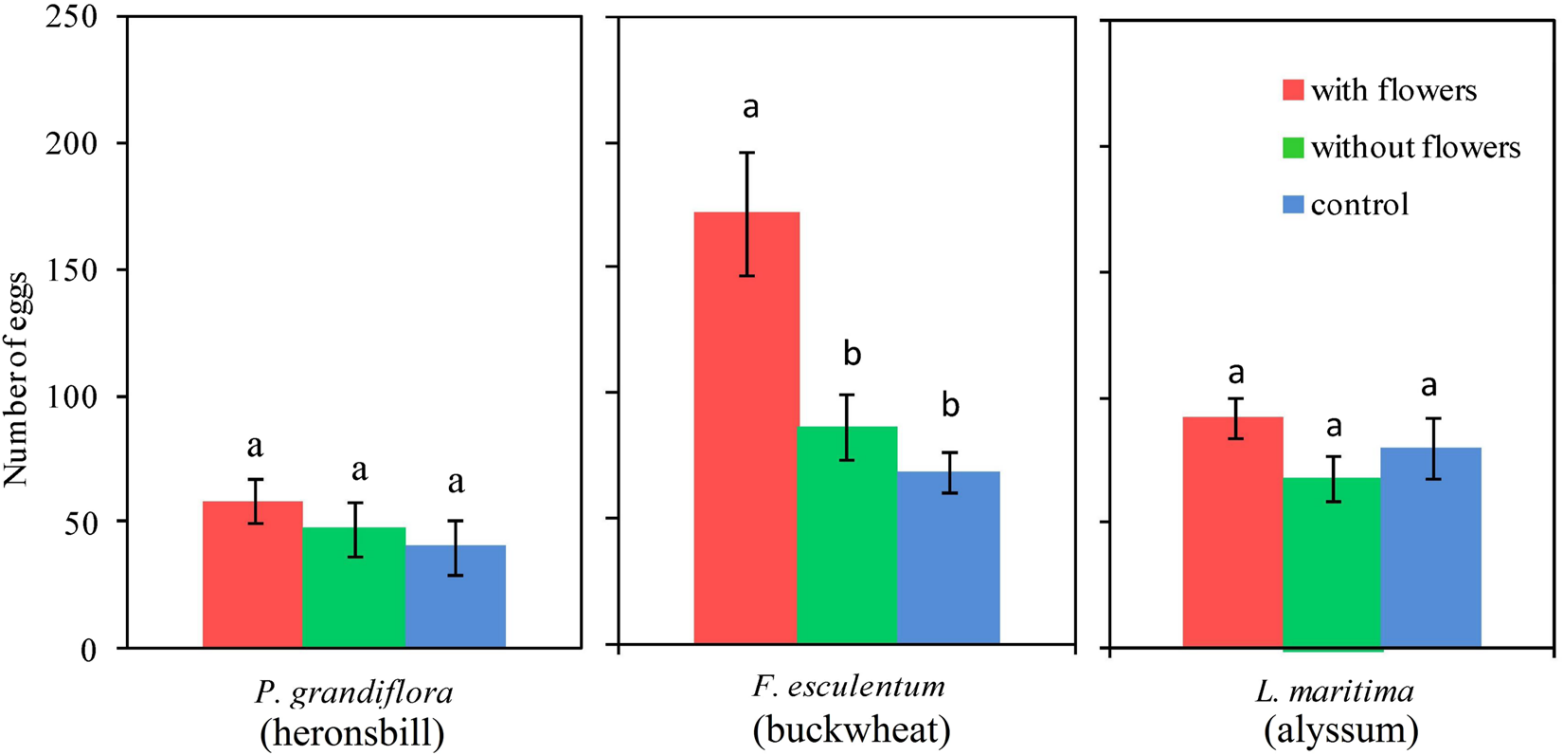
The fecundity of *P. xylostella* provided with three flowering plants treatments determined by the total number of eggs laid over life time. Treatments within flowering plant species labeled with the same letter are not significantly different (ANOVA, α < 0.05), error bars = ±1 SE (one pair of *P. xylostella* adults in each treatment, 9 replications).

In agreement with our longevity results, only buckwheat increased the lifetime fecundity of *P. xylostella* compared with plants without flowers and the water control (Welch ANOVA: F = 7.893, *P* = 0.005; Fig. 3). There were no significant effects of heronsbill flowers (one-way ANOVA: F_2, 26_ = 0.795, *P* = 0.463) and alyssum flowers (one-way ANOVA: F_2,26_ = 1.420, *P* = 0.261) on the realized lifetime fecundity of *P. xylostella* (Fig. 3).

### Behavioral responses of female *C. vestalis* to flowering plants

**Figure 4:**
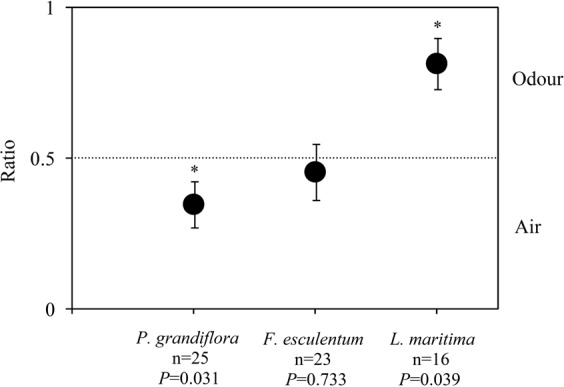
Ratio of the total time *C. vestalis* spent in the distal zone of the odour arm over the total time spent in distal zones of odour and air arms. A ratio of 0.5 indicates no preference; a ratio over 0.5 indicates a preference for the odour; a ratio below 0.5 indicates a repellence by the odour. Asterisks indicate a significant deviation from 0.5 (Wilcoxon signed rank test, P < 0.05), error bars = ±1 SE.

The Y-tube olfactometer study showed that parasitoids were significantly attracted to alyssum (Z = −1.705, *P* = 0.039), but not buckwheat (Z = −0.341, *P* = 0.733) (Fig. 4). Further, *C. vestalis* was significantly repelled by volatiles from heronsbill (Z = −2.158, *P* = 0.031) (Fig. 4).

## Discussion

Selectively attracting and enhancing the performance of parasitoids without benefiting pests is a crucial factor in screening flowering plants in the laboratory before moving to field evaluation for conservation biological control^9,54^. In this study, alyssum selectively attracted and enhanced the performance of *C. vestalis*. In the longevity experiments, the three flower species tested increased the lifespan of *C. vestalis*. This coincides with other studies concerning the impact of buckwheat and alyssum on survival of natural enemies^6,55^. The longevity of *C. vestalis* females was markedly greater than males in all diet treatments. Similar results have been reported for several other parasitoids, such as *Diadegma insulare* (Cresson) (Hymenoptera: Ichneumonidae)^56^ and *Microplitis croceipes* (Cresson) (Hymenoptera: Braconidae)^57^. Longer lifespan of female parasitoids may be because they need more time to mature and oviposit eggs and locate hosts than males^48^.

In our study, both buckwheat and alyssum flowers improved the fecundity of *C. vestalis*. The nutrients from flowers may support *C. vestalis* to mature more eggs for parasitism. Floral nectar provides not only sugars for parasitoids, but also proteins, amino acids and lipids^58–60^, which are vital for fecundity of parasitoids^61,62^. However, the fecundity of female *C. vestalis* was not improved by heronsbill. This may have occurred as a result of the repellency shown by heronsbill in the olfactometery study.

Alyssum and heronsbill flowers did not benefit *P. xylostella* longevity or fecundity. While buckwheat enhanced the performance of *C. vestalis*, it also improved the longevity and fecundity of *P. xylostella* indicating it may not be suitable to plant in agroecosystems where this pest is a major threat. This result is in line with previous studies^7,54^, although Lavandero *et al*.^54^ showed no significant positive effect of buckwheat on fecundity of *P. xylostella* under laboratory conditions. The reason could be that the eggs laid on the wall of cages as well as the plants were all counted in our study, but only eggs on the plant leaf were counted in previous studies^54^. Whilst the present study showed that buckwheat flowers improved the fecundity of the insect pest *P. xylostella*, this may not necessarily render it as being completely unsuitable for use in conservation biological control. Indeed, field research has shown that borders composed of buckwheat did not increase *P. xylostella* larval and pupal densities^63^.

This apparent conflict may reflect that any potential benefit provided by the nectar of buckwheat to *P. xylostella* that led to aggregation and higher local egg production could be offset by increased attack by multiple species of natural enemy that are also attracted to buckwheat. For example, in an organic cabbage agro-ecosystem, in the presence of buckwheat and/or alyssum, the abundance of the predatory coccinellid beetle *Coleomegilla maculata* De Geer (Coleoptera: Coccinellidae), a key predator of *P. xylostella* eggs and larvae, was significantly increased^64^. In our studies the effects of nectar plants on *C. vestalis* and *P. xylostella* were tested in isolation so, whilst caution seems to be necessary in the use of buckwheat, future testing should not entirely discount its potential value, especially given that *P. xylostella* is not the only economic pest of brassicas.

The response of *C. vestalis* to odours of buckwheat and heronsbill did not correlate with the performance of this parasitoid when caged under non-choice conditions on these two flowering plants. This is consistent with our hypothesis that the attractiveness of flowering plants do not necessarily correlate with their potential value to the parasitoid. This hypothesis has been tested for other parasitoid species of several lepidopteran pests, such as *M. mediator*^24,65^, *Cotesia glomerata* (L.) (Hymenoptera: Braconidae), and *Pimpla turionellae* (L.) (Hymenoptera: Ichneumonidae) with contrasting results among studies^25^. The present result indicates that providing buckwheat or heronsbill in an agroecosystem (even on the basis of increased longevity and fecundity of *C. vestalis*) may not ensure nectar feeding because *C. vestalis* adults may not be attracted to it.

Several studies have shown that *C. vestalis* was attracted by crucifer plants infested by *P. xylostella* because of the herbivore-induced plant volatiles (HIPVs) emitted from the plants^66,67^. Alyssum also is a cruciferous plant and has been found to be a good candidate for use as a trap crop for *P. xylostella* because it was highly attractive for *P. xylostella* to visit^68,69^ and oviposit but unsuitable for larval development^70^. When trap crops (glossy collards (*Brassica oleracea* var. italica), kale (*Brassica oleracea* var. acephala) and mustard (*Brassica juncea*)) and insectary plants (buckwheat and alyssum) were planted in an organic cabbage system, higher numbers of *P. xylostella* adults on trap crops adjacent to insectary plants could be due to high levels of attraction of alyssum to *P. xylostella*^64^. One way to enhance the efficiency of natural enemies is by combining a synthetically-produced HIPV (“attract”) with a floral resource (“reward”), a concept termed “attract and reward”^71–73^. Combining methyl salicylate (MeSA) and the floral resource buckwheat could be beneficial because they increase the abundance of different natural enemies^74^. Further laboratory and field tests are needed to determine whether the HIPVs emitted by alyssum may have multiple attractiveness to *C. vestalis*.

Synovigenic parasitoids not only search for hosts, but also for plant foods – principally nectar – to obtain energy and nutrients for egg maturation and energy for foraging^50^. Providing plant foods in field for parasitoids saves the traveling time searching for supplementary foods^75^, and also promotes the parasitoid aggregation in host patches, which could improve parasitism rates^76^. Our results show that alyssum may be a particularly useful and selective food plant to enhance the performance of *C. vestalis* in the field, due to its positive effect on the performance of the beneficial, *C. vestalis* but not on the pest, *P. xylostella*. Alyssum alone or combined with buckwheat also could enhance the abundance of other natural enemies^64,77,78^ and attractiveness of nectar plants, combined with the chemical signs from hosts, could prolong the residence period of parasitoids in host patches^76^. Further field work is now important to measure the ultimate level of *P. xylostella* control and effects on other pests and natural enemies.

## Methods

### Experimental conditions

All experiments were conducted in a climate chamber at 25 ± 2 °C, 50 ± 10% RH and a photoperiod of 14:10 h (L:D). Flowering heronsbill, buckwheat and alyssum seeds were purchased from Jianxin Flower Market, Fuzhou, Fujian Province, China. Buckwheat and alyssum seeds were individually sown in plastic pots. Seedlings were kept in the greenhouse for 15 days, before individual seedling was transferred into plastic pots (20 cm diameter, 15 cm height) outdoors. Plants were checked and watered on a daily basis and fertilized at a rate of 10 mL/3 L every two weeks using plant nutrient solution (Dewoduo Fertilizer Company, Hengshui, Hebei, China) until the plants flowered. All three plant species were used in all of the assays.

The *P. xylostella* and *C. vestalis* strain used in our study were established several years ago from field population in Fuzhou, Fujian, China. *Plutella xylostella* larvae were reared on 5-days-old radish plants (*Raphanus sativus* L.) in cages which were 50 × 50 × 50 cm in size and covered with a fine metal mesh. Twenty pairs of adult *P. xylostella* were introduced into a cage which contained about 5-day-old radish plants for oviposition. After 24 h, the tray was moved to another cage for keeping most larvae at the same instar. The plants were watered as necessary. The larvae were transferred to fresh plants using a soft brush before the leaf material was depleted. Pupae were collected and transferred to individual Eppendorf tubes.

*Cotesia vestalis* were reared on *P. xylostella* larvae. About 30 pairs of adult *C. vestalis* were introduced into the cage when the *P. xylostella* larvae were second to third instar for parasitism. Diluted honey (10%) was provided as a food supplement for the adult parasitoid. About after two weeks, parasitoid cocoons were collected and individually placed in Eppendorf tubes for experiments.

### Effect of flowering plants on *C. vestalis* adult longevity and fecundity

For the longevity and fecundity experiment, we used cages (30 × 30 × 50 cm) covered with mesh. Three treatments: (1) plants with flowers plus water, (2) plants without buds or flowers (any buds were removed by hand 2 days before the assay) plus water, (3) pot and soil plus water only (control). A moistened piece of cotton was placed on the top of cage to provide water daily. The plants in cages were watered daily. Flowering plant species were not tested concurrently but identical environmental conditions were used across studies. Each treatment was replicated 9 times. A pair of *C. vestalis* adults, newly emerged (within 6 h) that had not been provided any food or water was provided in each cage. Fecundity was assessed for *C. vestalis* during the same experiment used to monitor longevity. A petri dish with 30 *P. xylostella* larvae (2-3 instar) and radish sprouts was also provided in each cage for the fecundity assay. The roots of radish sprouts were wrapped with a wet piece of cotton to prevent wilting. The *P. xylostella* larvae were renewed every 24 h until the female adult of *C. vestalis* died. The *P. xylostella* larvae were removed from cages and reared with fresh radish sprouts until *C. vestalis* larvae emerged from the host and spun a cocoon or the *P. xylostella* pupated. Adult survival of *C. vestalis* was recorded at 12 h intervals until all had died. Pupae of *C. vestalis* and *P. xylostella* were counted every 24 h. Parasitism rates were calculated as ratio of pupae of *C. vestalis* and total pupae (included *C. vestalis* and *P. xylostella*).

### Effect of flowering plants on *P. xylostella* adult longevity and fecundity

**Figure 5:**
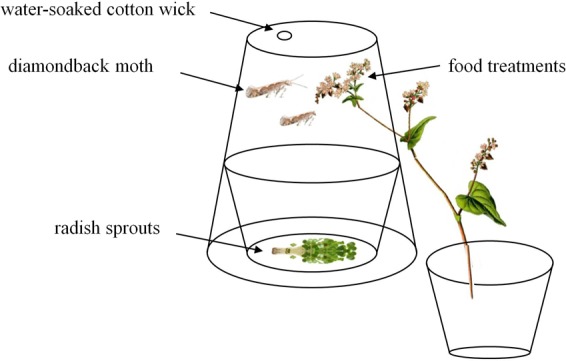
Experimental setup. Cages constructed from clear plastic container used to house *P. xylostella* adult for longevity and fecundity studies.

Small cages to house *P. xylostella* were constructed using clear plastic containers (8 cm top diameter, 12 cm bottom diameter, 15 cm height) with several small pinholes on the walls to provide ventilation. A small slit lengthwise (10 cm) was cut on the wall to allow insertion of flowers. A hole of about 1 cm diameter was cut on the top and plugged with a water-soaked cotton wick (Fig. 5).

The longevity and fecundity of *P. xylostella* were investigated when provided the following three treatments as food: (1) plant stem with flowers, (2) plant stem without flowers, and (3) water only. All treatments were provided with free water. Each plant species was tested separately but environmental conditions were consistent. A pair of newly emerged (<24 h) unfed and unmated *P. xylostella* adults were introduced into each cage. Radish sprouts whose roots were wrapped with wet cotton wool were placed on a Petri dish on the base of the cage for oviposition and replaced daily. Adult *P. xylostella* survival was recorded every 12 h until all individuals died. Mean survival time was then calculated. In addition, the number of eggs laid on the radish sprouts and cages were recorded daily to assess realized lifetime fecundity. The assay was replicated 9 times for each plant species.

### Attractiveness of flowering plant to *C. vestalis*

Attractiveness experiments used a dynamic airflow Y-tube olfactometer for choice experiments. The Y-tube had a 2 cm inner diameter with a 13 cm-long central tube and two 11.5 cm-long side arms (Shitang Glass, Hangzhou, Zhejiang Province, China). The angle between the two arms was 60°. Two arms were in turn connected to a glass chamber (22 cm diameter, 47 cm height) which held the plants, through a tube. The olfactometer was placed in a 30 × 30 × 30 cm cage in a dark room with a single 11-watt light bulb overhead to avoid directional light. The experiments were conducted between 10:00 and 14:00 when *C. vestalis* is most active. The air was pushed by a vacuum pump through the olfactometer, filtered on allochroic silica gel, activated charcoal and a molecular sieve (Sigma, USA) to avoid impurities from ambient air. The airflow was kept at 400 ml min^−1^ controlled by flow meters. Whole flowering plants of heronsbill, buckwheat and alyssum were used as the odour source, i.e. treatments and compared with clean air in each case. The plant pots were wrapped with tinfoil before they were placed into a container. Thirty *C. vestalis* female adults were tested in each treatment. Adult *C. vestalis* were transferred from rearing cages to a 2 ml Eppendorf tube for 2 h before testing without food. Individual female *C. vestalis* was released at the entrance of the olfactometer. The time that parasitoid spent in the distal zone was recorded as residence time during 5 minutes. The distal zone was between the fine mesh at end of the arm and a line (4 cm before the end of the arm). If the parasitoid did not cross the start line (2 cm before the fond end of the central tube) within 2 minutes, they were considered non-responders and removed from the analysis. The location of the treatment or the control, i.e. in the left or right arms of the olfactometer was switched every 5 replications. The olfactometer was cleaned with 95% ethanol and dried every 10 replications.

### Statistical analysis

Prior to analysis, the Shapiro-Wilk test was used to test for the normality of data. When data did not fit a normal distribution, square-root or log 10 transformations was used. When the assumption of normality was fulfilled, a one-way ANOVA or Welch ANOVA was used to test the effects of flowering plant treatments on female and male longevity and fecundity of parasitoid and *P. xylostella*. When no transformation was able to normalize the variance, the Kruskal-Wallis test was applied. Kaplan – Meier estimator was used to do a survival analysis to compare the effects of flowering plants on the longevity of *C. vestalis* and *P. xylostella*. Wilcoxon signed-rank test was performed to test whether there was a significant preference by female *C. vestalis* for two arms because the data did not conform to normal distribution. SPSS version 19.0.0 was used to perform the computations.
